# Regulatory T cells induced by B cells: a novel subpopulation of regulatory T cells

**DOI:** 10.1186/s12929-017-0391-3

**Published:** 2017-11-18

**Authors:** Chien-Hui Chien, Bor-Luen Chiang

**Affiliations:** 1 Graduate Institute of Clinical Medicine, National Taiwan University, Taipei City, 10048 Taiwan, Republic of China; 2 Department of Medical Research, National Taiwan University Hospital, Taipei City, 10002 Taiwan, Republic of China

**Keywords:** Regulatory T cells, Lymphocyte-activation gene 3, Programmed cell death protein 1, Inducible T-cell co-stimulator, Interleukin 10, Cytotoxic T lymphocyte-associated antigen-4, Treg-of-B cells

## Abstract

Regulatory T cells play a crucial role in the homeostasis of the immune response. In addition to CD4^+^Foxp3^+^ regulatory T cells, several subsets of Foxp3^-^ regulatory T cells, such as T helper 3 (Th3) cells and type 1 regulatory T (Tr1) cells, have been described in mice and human. Accumulating evidence shows that naïve B cells contribute to tolerance and are able to promote regulatory T cell differentiation. Naïve B cells can convert CD4^+^CD25^-^ T cells into CD25^+^Foxp3^-^ regulatory T cells, named Treg-of-B cells by our group. Treg-of-B cells express LAG3, ICOS, GITR, OX40, PD1, and CTLA4 and secrete IL-10. Intriguingly, B-T cell-cell contact but not IL-10 is essential for Treg-of-B cells induction. Moreover, Treg-of-B cells possess both IL-10-dependent and IL-10-independent inhibitory functions. Treg-of-B cells exert suppressive activities in antigen-specific and non-antigen-specific manners in vitro and in vivo. Here, we review the phenotype and function of Foxp3^+^ regulatory T cells, Th3 cells, Tr1 cells, and Treg-of-B cells.

## Background

Regulatory T cells are a therapeutic strategy for immune dysregulated diseases and a potential target for cancer immunotherapy. In addition to CD4^+^Foxp3^+^ regulatory T (Treg) cells, studies have emphasized the roles of CD4^+^Foxp3^-^ regulatory T cells, such as TGF-β-producing T helper 3 (Th3) cells, IL-10-producing type 1 regulatory T (Tr1) cells, and others. Accumulating evidence demonstrate that naïve B cells possess the ability to promote naïve CD4^+^ T cells into CD25^+^ Foxp3^-^ regulatory T cells with the expression of lymphocyte activation gene-3 (LAG3, CD223), inducible co-stimulator (ICOS, CD278), programmed cell death protein 1 (PD1, CD279), and glucocorticoid-induced TNFR family-related protein (GITR). B-cell-induced CD4^+^Foxp3^-^ regulatory T cells exert the inhibition through both IL-10-independent and cell-cell contact-dependent mechanisms, although they also show IL-10-mediated suppression. Furthermore, these B cell-induced regulatory T cells protect mice from several immune disorders, including graft-versus-host disease, experimental allergic asthma, collagen-induced arthritis, and inflammatory bowel disease. Here, we review the phenotypes and functional mechanisms of thymus-derived and peripherally derived CD4^+^Foxp3^+^ regulatory T cells, Th3 cells, Tr1 cells, B-cell-induced Foxp3^-^ regulatory T cells, and B-cell-induced Foxp3^+^ regulatory T cells. The present article focuses on B-cell-induced CD4^+^Foxp3^-^ regulatory T cells, which we have named Treg-of-B cells.

## Main text

### CD4^+^Foxp3^+^ regulatory T cells

Sakaguchi et al. demonstrated that CD4^+^CD25^+^ T cells contributed to maintaining self-tolerance in a non-antigen-specific manner [1]. Immune dysregulation, polyendocrinophathy, enteropathy X-linked (IPEX) syndrome is a recessive immune disorder. Reports showed that IPEX is caused by mutations of *FOXP3* gene, which is orthologouse of the *Foxp3* gene mutated in scurfy mouse [2–4]. Further studies demonstrated that Foxp3 expressed predominantly in CD4^+^CD25^+^ T cells than CD4^+^CD25^-^ T and CD19^+^ B cells. Moreover, retroviral transduction of Foxp3 in naïve CD4^+^CD25^-^ T cells converted these cells toward Treg cells phenotype. Thus, Foxp3 has been identified as the master transcription factor of Treg cells [5].

### Thymus-derived Foxp3^+^ regulatory T cells

In addition to Foxp3, thymus-derived CD4^+^CD25^+^Foxp3^+^ regulatory T (tTreg) cells highly expressed Helios, cytotoxic T lymphocyte-associated antigen-4 (CTLA4, CD152), neuropilin-1, GITR, galectin-1, IL-10, and granzyme B [6]. tTreg cells could be activated in an antigen-specific fashion and exerted suppressive activity in a non-antigen-specific fashion [7]. tTreg cells produced many inhibitory cytokines, including TGF-β1, IL-10, and IL-35, to downregulate immune responses [8]. Furthermore, tTreg cells exhibited cell-cell contact-dependent suppression via latency-associated peptide (LAP) [9], CD39 (ectonucleoside triphosphate diphosphohydrolase-1, ENTPD1) and CD73 (ecto-5′-nucleotidase) [10], and cytosolic cyclic adenosine monophosphate (cAMP) [11]. Reports showed that tTreg cells induced effector T cell apoptosis via various pathways, including deprivation of IL-2 and IL-7 [12], disruption of effector cell membrane integrity by granzyme B [13], galectin-1-induced apoptosis [14], and the engagement of TNF-related apoptosis inducing ligand (TRAIL)-death receptor 5 (DR5) [15]. Additionally, tTreg cells inhibited effector T cell activation via downregulation of costimulatory molecules on DCs through CTLA4 [16] and LAG3 [17]. These studies indicate that tTreg cells are a polyclonal population, and the above mentioned complicated mechanisms result in maximal immunosuppression during homeostasis.

### Peripherally derived Foxp3^+^ regulatory T cells

Foxp3^+^ regulatory T cells induced in vivo are called peripherally derived regulatory T (pTreg) cells and those generated in vitro are called in vitro-induced regulatory T (iTreg) cells [18]. Studies demonstrated that CD4^+^Foxp3^-^ T cells differentiated into Foxp3^+^CD25^+^CD45RB^low^ anergic T cells with suppressive functions in the presence of TGF-β1 in vitro as well as in vivo [19] and rescue Foxp3-deficient scurfy mice [20]. In the absence of tTreg cells, oral antigen administration induced the generation of CD4^+^CD25^+^Foxp3^+^ regulatory T cells in a TGF-β1-dependent manner [21]. Gut-associated lymphoid tissue CD103^+^ DCs played an important role in the *de novo* conversion of naïve T cells into pTreg cells, and retinoic acid facilitates that process [22]. Additionally, lung-resident tissue macrophages expressed retinal dehydrogenases, and TGF-β1 promoted pTreg cell induction under steady-state conditions [23]. Evidence has shown that the tumor environment induced pTreg cell generation to escape immune clearance [24]. One report demonstrated that tTreg and pTreg cells shared similar phenotypes, and neuropilin-1 serving as a surface marker to distinguish tTreg cells from pTreg cells [25].

### CD4^+^Foxp3^-^ regulatory T cells

The most well-defined Foxp3^-^ regulatory T cells are Th3 cells and Tr1 cells. Th3 cells have been identified as TGF-β-producing CD4^+^LAP^+^ T cells exhibiting TGF-β-mediated suppression [26]. Tr1 cells have been characterized by the higher production of IL-10 and IL-10-mediated suppressive functions [27].

### T helper 3 cellsl

Th3 cells were first found in mesenteric lymph node CD4^+^ T cells as single cell clones producing TGF-β1 after oral administration of self-antigen [28]. Oida et al. found that primary purified CD4^+^CD25^-^LAP^+^ regulatory T cells protected mice from T-cell-induced colitis in a TGF-β1-dependent manner [29]. Tumor environment CD4^+^CD25^-^CD69^+^Foxp3^-^LAP^+^ T cells expressed IL-2 receptor β chain, produced TGF-β1, and exerted TGF-β1-mediated functional activity [30]. Gandhi et al. showed that human peripheral CD4^+^LAP^+^Foxp3^-^CD69^+^ T cells exhibited TGF-β1- and IL-10-dependent suppression in the periphery in healthy individuals [31]. Furthermore, human CD4^+^CD25^+^LAP^+^Foxp3^-^ T cells in colorectal tumors expressed LAG3 and exhibited inhibitory functions through TGF-β1 and IL-10 [32]. To date, the specific transcription factor for Th3 cells remains to be identified.

### Type 1 regulatory T cells

The first study on Tr1 cells reported that naïve T cells repeated stimulation with peptide-pulsed splenocytes in the presence of IL-10 induced IL-10-producing CD4^+^ T cells with suppressive ability and hypoproliferative ability [33]. Akbari et al. demonstrated that bronchial DCs promoted Tr1 cells in vitro in an IL-10-and ICOS/ ICOS ligand (ICOSL)-dependent manner in the context of nasal tolerance [34]. By microarray analysis Tr1 and Th0 cell clones, CD49b, LAG3, and CD226 have been identified as the surface markers of Tr1 cells [35].

It has been shown that c-Maf transactivated IL-10 expression under CD4^+^ Th17 polarization conditions [36]. Aryl hydrocarbon receptor (AhR) and c-Maf facilitated IL-10 production in CD4^+^ T cells in an IL-27-dependent fashion [37, 38]. Another study reported that c-Maf, IL-21, and ICOS were essential for IL-27-induced Tr1 cell generation [39]. Consistent with these observations, Awasthi et al. showed that CD4^+^Foxp3^+^ regulatory T cell-educated DCs produced IL-27 and promoted Tr1 cell generation [38]. Nasal anti-CD3ɛ antibody treatment induced the expression of IL-10, IL-27, and TGF-β in nasal tolerogenic DCs, which further facilitated Tr1 cell generation through c-Maf, IL-21, and AhR [40]. Orally antigen treated tolerogenic Peyer’s patch DCs increased the production of IL-10 and IL-27 and promoted the induction of Tr1 cells [41]. Carrier et al. reported that constitutive ectopic expression of GITR ligand (GITRL) on MHCII^+^ APCs increased IL-27 production and further upregulated the expression of c-Maf and IL-10 in T cells [42].

In addition to cytokines, reports have demonstrated that Tr1 cells could be induced by different proteins, different APCs, and different types of T cells. Galectin-1 promoted IL-10 expression in CD4^+^ T cells in an APC-independent pathway by binding to CD45 on T cells and inducing the expression of c-Maf and AhR [43]. In vitro activation of CD4^+^CD44^hi^Foxp3^-^ T cells through anti-CD3/CD28 antibodies and IL-2 generated CD49b-, LAG3-, c-Maf-, and AhR-expressing Tr1 cells [44]. Nie et al. found that long-term stimulation of lipopolysaccharide (LPS) conferred ICOSL expression in bone marrow-derived mast cells through NF-κB, subsequently promoting Tr1 cell development [45]. These reports suggest that the generation mechanisms for Tr1 cells consist of a fine-tuning program.

### B cells in tolerance induction

B cells have been shown to have a role in the fine equilibrium for immune tolerance. Genetically B-cell-deficient mice delayed recovery from experimental autoimmune encephalomyelitis and suggested B cells might contribute to immune modulation [46]. Collagen fragments expressed on B cell MHC class II sufficiently delayed the onset and decreased the severity of arthritis [47]. The role of B cells in oral tolerance has been investigated because B-cell-deficient mice exhibit a defective oral tolerogenic response characterized by lower levels of IL-10 and TGF-β in the spleen and gut-associated lymphoid tissues [48]. Gutgemann et al. showed that B cells interacted with T cells at the B-T border in the spleen after 4 h of oral administration of proteins [49]. Furthermore, orally antigen treated B cells have an enhanced ability to induce CD4^+^ regulatory T cells in vitro [50]. Anterior chamber-associated immune deviation was characterized by antigen-specific downregulation of the immune response to antigen occurs in the anterior chamber of the eye [51], and this phenomenon was abrogated in the absence of B cells [52]. Studies suggested that splenic B cells presented antigens derived from ocular APCs and induced CD4^+^CD25^+^ regulatory T cells via IL-10 and MHC class II [52, 53]. These evidence emphasize the role of B cells in the induction and maintenance of self-tolerance.

There is accumulating evidence demonstrating that specific B cell subsets modulate immune responses named as regulatory B (Breg) cells by Mizoguchi et al. [54]. Breg cells dampened immune responses though the secretion of IL-10, TGF-β, directly interact with activated CD4^+^ T cells, and the production of antibody that neutralized harmful soluble molecules [55]. Several Breg cells have been described in mice and IL-10-producing Breg cells are the most widely studied [56]. IL-10 produced by a variety of Breg cells suppressed inflammatory cytokines and promoted regulatory T cell differentiation [57, 58]. These indicate that B cells contribute to the maintenance of tolerance.

In addition, naïve B cells functioned as antigen-presenting cells presented antigen and resulted in T cell tolerance to antigen [59]. Raimondi et al. demonstrated that adoptive transfer of antigen-presenting B cells four times in a week lead to antigen-specific CD4^+^ T cells tolerance independent of naïve or activated B cells [60, 61]. Antigen-presenting follicular B, marginal zone B, and B-1a cells rendered antigen-specific T cells hyporesponsiveness without Foxp3^+^ Treg cells induction [62]. One study reported that B cells contributed to Treg cells homeostasis and cooperated with Treg cells to ameliorate inflammation [63]. These findings suggest that B cells play a role in immune modulation and might through the manipulation of CD4^+^ Treg cells.

### B-cell-induced CD4^+^Foxp3^-^ Treg-of-B cells

Naïve splenic B2 cells, peritoneal B-1a cells, and mucosal Peyer’s patch B cells have been shown to induce CD4^+^CD25^+^Foxp3^-^ regulatory T cells, which named Treg-of-B cells by our group, without additional cytokines or molecules [50, 64]. Naïve splenic B cells and naïve splenic CD4^+^CD25^-^ T cells formed a stable immunological synapse and promoted CD62L^hi^CD25^+^Foxp3^-^ regulatory T cell generation [65]. In our reports, transwell insertion during B-T coculture abrogated Treg-of-B cell induction suggesting that cell-cell contact between B and T cells was essential. By applying blocking antibodies during B-T coculture, both CD80 and CD86 on splenic B cells were required to induce functional Treg-of-B [64]. In consistent with above, Etemire et al. demonstrated that addition of anti-CD28 antibody to the B-T cell co-culture decreased the suppressive activity of Treg-of-B cells. Lower activity of the PI3K/AKT pathway was associated with Foxp3^-^ regulatory T cell generation [66]. IL-10-deficient Treg-of-B cells and Treg-of-B cells induced in the presence of anti-IL-10 neutralizing antibody remained their suppressive function suggesting that IL-10 was not critical for their induction [64, 67, 68]. These results suggest that the interaction between B-T cells is indispensable for the differentiation of Treg-of-B cells.

### Treg-of-B cells differ from well-known Treg cells

**Fig. 1:**
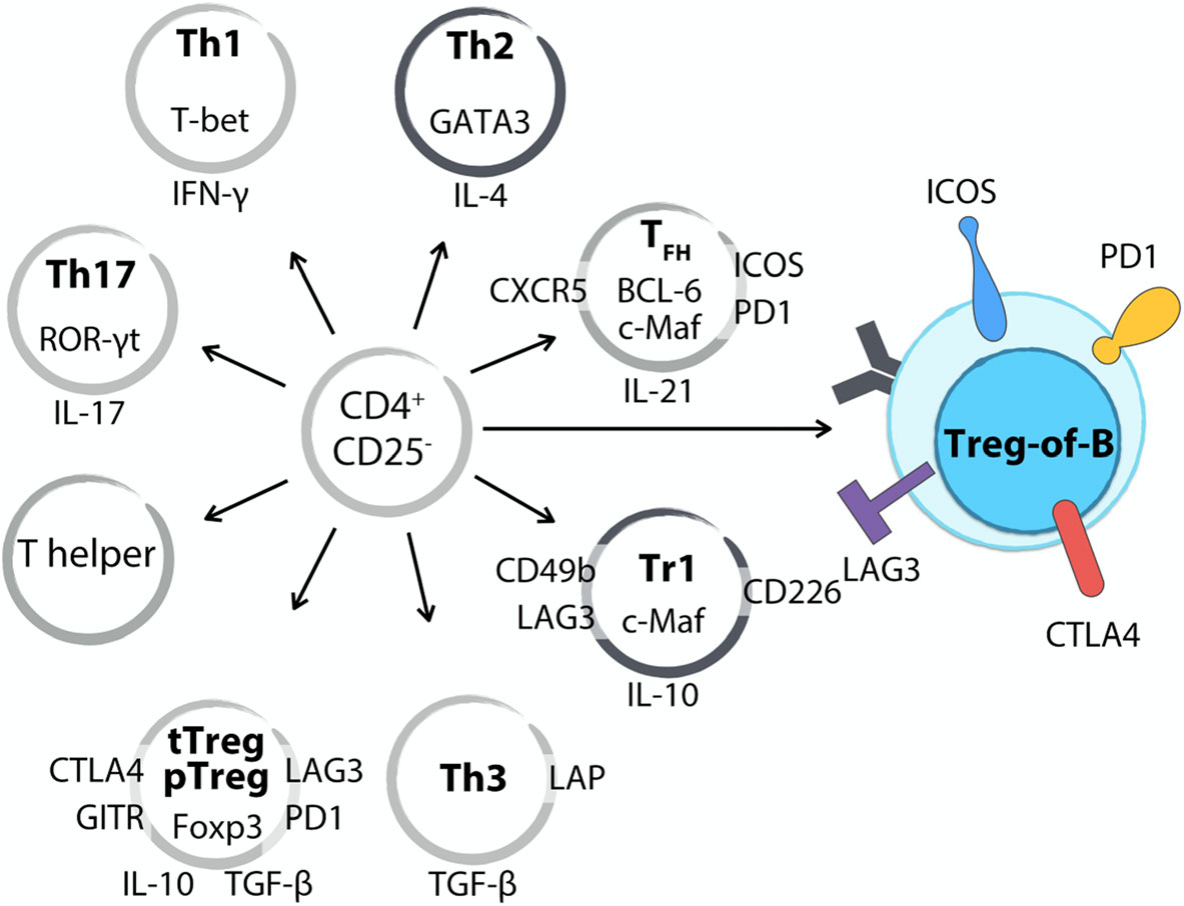
Treg-of-B cells differ from well-known regulatory T cells and T helper cells. With regard to transcription factors, Treg-of-B cells do not express Foxp3, ROR-γt, T-bet, or BCL-6. Repeated stimulation increased the expression of c-Maf in long-term Treg-of-B cells. Treg-of-B cells produce a higher amount of IL-10 and TGF-β and lower amounts of IL-17 and IFN-γ. Several Treg-associated molecules have been described in Treg-of-B cells, including LAG3, PD1, ICOS, CTLA4, and GITR. Long-term cultured Treg-of-B cells express CD49b but do not express CD226 as Tr1 cells. Treg-of-B cells do not express ROR-γt as Th17 cells do, do not express T-bet as Th1 cells do, do not express CXCR5 or BCL-6 as T_FH_ cells do, and do not express LAP as Th3 cells do. These indicate Treg-of-B cell is a new type of CD4^+^ regulatory T cells

To date, several molecules have been identified for their strong association with Treg-of-B cells that are conserved in single peptide-induced and anti-CD3/CD28 antibodies-induced methods. Treg-of-B cells expressed higher levels of LAG3, ICOS, PD1, GITR, OX40 (CD134), and CTLA4 compared to those on naïve CD4^+^CD25^-^ T cells (Fig. 1). Another group demonstrated that antigen-presenting B cells facilitated naïve T cells to convert into CD4^+^CD25^+^CD62L^+^Foxp3^-^ IL-10-producing regulatory T cells [65]. Our published and unpublished data showed that Treg-of-B cells did not express Foxp3, Helios, or neuropilin-1 [67, 69], and these also confirmed by using Foxp3-GFP reporter mice [64]. These evidence differentiates Treg-of-B cells from Foxp3-expressing Treg cells (Table 1).

**Table 1:** The differences between Treg-of-B cells and the well-known Treg cells, including Foxp3^+^ Treg, Th3, and Tr1 cells

Treg cells	Biomarkers	Effector molecules	Transcription factors	Assisted cell types
Treg-of-B	CD4^+^CD25^+^Foxp3^-^LAG3^+^ICOS^+^PD1^+^GITR^+^OX40^+^	Majorly contact-dependent IL-10, LAG3, and CTLA4 has reported in reference	Undefined	B cells
Foxp3^+^ Treg	CD4^+^Foxp3^+^ Helios has reported in reference	IL-10, TGF-β, IL-35, LAP, CD39/CD73, cAMP, CTLA4, LAG3, IL-2/IL-7 consumption, granzyme B, galectin-1, DR5…etc	Foxp3	DCs, macrophages, B cells
Th3	CD4^+^Foxp3^-^LAP^+^	Majorly TGF-β IL-10 has reported in reference	Undefined	DCs
Tr1	CD4^+^Foxp3^-^CD49b^+^LAG3^+^ CD226^+^	Majorly IL-10 TGF-β, CTLA4, and CD226 has reported in reference	Undefined c-Maf, AhR has reported in reference	DCs, macrophages, B cells, mast cells…etc

Th3 cells are well-known that they exert TGF-β-dependent inhibition and express LAP on surface [26]. Although Treg-of-B cells produced TGF-β compared with naïve CD4^+^CD25^-^ T cells [68, 69], TGF-β did not play a role in their suppressive mechanism [64]. In our unpublished data, Treg-of-B cells did not express LAP. These indicate that Treg-of-B cells are different from Th3 cells.

Tr1 cells are characterized by IL-10-mediated suppression and the higher production of IL-10 [27]. In recent years, CD49b, LAG3, and CD226 were identified as the surface markers for human and mouse Tr1 cells [35]. In our results, Treg-of-B cells produced a higher amount of IL-10 compared with naïve CD4^+^CD25^-^ T cells [50, 64]. Repeated stimulation of B cells induced long-term Treg-of-B cells with higher expression of ICOS, CTLA4, CD49b, and c-Maf, but not CD226. In addition to the difference in surface marker, IL-10 seems to be dispensable in the inhibitory mechanism of Treg-of-B cells and these would be described in the later section. These observations suggest that this Treg-of-B cell is a new type of regulatory T cells and different from Tr1 cells.

In addition to regulatory T cells, Treg-of-B cells did not share characteristics with follicular T helper (T_FH_) cells. T_FH_ cells expressed BCL-6, CXCR5, ICOS, PD1, and c-Maf and CXCR5 conferred T_FH_ cells migration to B follicles [67, 70]. Although Treg-of-B cells expressed ICOS, PD1 and c-Maf, they did not express the critical molecule BCL-6 and CXCR5 (data not shown). These indicate that Treg-of-B cells could not migrate into follicle to facilitate B cell as T_FH_ cells did.

Furthermore, Treg-of-B cells were hypoproliferative to stimulation and did not express T-bet, GATA3, or ROR-γt ([64] and our unpublished data). Treg-of-B cells produced higher level of IL-10, TGF-β, and IL-4 and lower or no IL-2, IFN-γ, IL-17, or tumor necrosis factor (TNF)-α [68, 69, 71]. These data confirm that Treg-of-B cells have anergic characteristics and are not proinflammatory T helper cells.

### Application of Treg-of-B cells

**Fig. 2:**
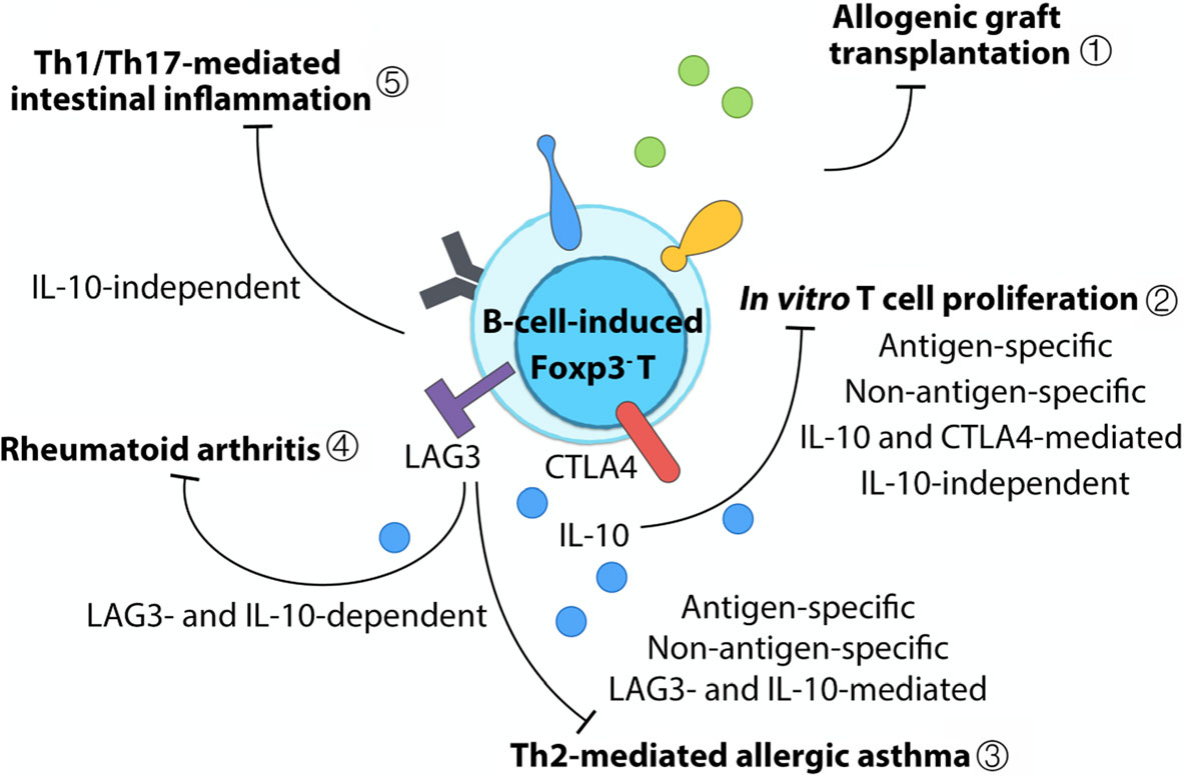
B-cell-induced CD4^+^Foxp3^-^ regulatory T cells treatment in disease models. Treg-of-B cells have been used for therapy in several animal models. Pre-treated Treg-of-B cells prevented allogeneic heart transplantation-induced tissue rejection ①. Treg-of-B cells inhibited antigen-specific and non-antigen-specific T cell proliferation in vitro through IL-10-mediated and IL-10-independent mechanisms. Both IL-10 and CTLA4 play roles in long-term Treg-of-B cells suppressive functions ②. In vivo treatment with Treg-of-B cells protected mice from Th2-mediated allergic asthma in an antigen-specific manner and in a non-antigen-specific fashion ③. Both LAG3 and IL-10 may play roles in the protection of mice from allergic asthma and rheumatoid arthritis ④. IL-10-deficient Treg-of-B cells prevented mice from T-cell-mediated intestinal inflammation ⑤

The therapeutic effects of CD4^+^Foxp3^-^ Treg-of-B cells has been described in several murine disease models (Fig. 2). Adoptive transfer of Treg-of-B cells prevented mice from graft-versus-host disease in a murine model of heart transplantation [65]. Peyer’s patch B-cell-induced ovalbumin (OVA)-specific Treg-of-B cells protected mice from Th2-cell-mediated airway hyperresponsiveness (AHR), airway inflammation, and IgE hyper-production in allergic asthma in an antigen-specific fashion [50]. In addition, splenic B-cell-induced OVA-specific Treg-of-B cells shared several characteristics with oral antigen administration activated CD4^+^CD25^+^ T cells, including elevated expression levels of ICOS, PD1, and CTLA4 and enhanced non-antigen-specific suppressive functions [69]. Monoclonal antibody-induced Treg-of-B cells prevented mice from osteolysis and joint inflammation in collagen-induced arthritis [71]. Prophylactic transfer of Treg-of-B cells also protected mice from T-cell-induced Th1- and Th17-dominant inflammatory bowel disease [68]. Taken together, naïve B cell without cytokines or chemical supplements is able to induce functional CD4^+^Foxp3^-^ regulatory T cells and that B-cell-induced regulatory T cells is an economical strategy for cellular therapy for different T-helper-cell-dominant inflammatory diseases.

### Treg-of-B cells possess both IL-10-dependent and IL-10-independent suppressive functions

IL-10 as an anti-inflammatory cytokine is an issue in Treg-of-B cells suppressive function. As described above, IL-10 does not play a crucial role in Treg-of-B cells differentiation. Chen and Chu et al. reported that LAG3^+^Treg-of-B cells produced higher amount of IL-10 and both IL-10 and LAG3 play the roles in their inhibitory mechanisms [71, 72]. Long-term Treg-of-B cells increased expression levels of CTLA4 and IL-10, both of which were involved in their suppressive functions [67]. IL-10-deficient mice were used to confirm the role of IL-10 in the regulation; however, IL-10-deficient Treg-of-B cells remained suppressive activities [64, 68]. IL-10 seems to be dispensable in the inhibitory mechanism of Treg-of-B cells. Although IL-10 plays a more important role in long-term Treg-of-B cells than in short-term Treg-of-B cells, three-day short-term culture is sufficient for the generation of Treg-of-B cells. These suggest that there might be unknown inhibitory factors in Treg-of-B cells suppressive functions.

Studies have demonstrated that ICOS controls IL-10 production and functional CTLA4 expression in Treg cells [73–75]. PD1 recruits SHP-1 and SHP-2 to intrinsically downregulate T cell receptor signaling, which maintains an anergic phenotype in Treg cells [76, 77]. Mouse Treg cells constitutively expressed GITR and OX40 and involved the tTreg cells development as well as their functions [78–80]. All regulatory-T-related molecules on Treg-of-B cells, including IL-10, TGF-β, LAG3, CTLA4, ICOS, PD1, GITR, and OX40, might confer partial suppressive activities to compensate for single blockage or neutralization. The critical molecules controlling Treg-of-B cell phenotype and regulatory mechanisms remain priorities for investigation. The inhibitory functions of Treg-of-B cell depend on the suppressive molecules on the surface or soluble mediators that require short distance.

### B-cell-induced CD4^+^Foxp3^+^ regulatory T cells

Reports have revealed the role of B cells in the development of Treg cells. Naïve primary B cells preferentially induced the expansion of allogenic CD4^+^Foxp3^+^ T cells rather than CD4^+^Foxp3^-^ T cells [81, 82]. Splenic B cells converted allogenic naïve T cells into Foxp3^+^ regulatory T cells in the presence of TGF-β and IL-2, and peritoneal B cells induce Th17 cells [83]. Human CD40-activated B cells induced the differentiation of CD25^+^Foxp3^+^CD62L^+^ regulatory T cells more efficiently than immature DCs [84, 85]. In contrast, reports demonstrated that murine CD40-activated B cells promoted CD4^+^ T cell proliferation and effector functions [86, 87]. Furthermore, the frequency of intrathymic B cells correlated with that of tTreg cells, and B cells colocalized with tTreg cells in the thymus [88, 89]. Intrathymic B cells expressed autoimmune regulator (Aire), increased the levels of MHC class II and CD80, and contributed to T cell negative selection for central T cell tolerance [90, 91]. Taken together, there are unknown criteria, such as MHC class II-TCR signaling, the B cell activation status, and different types of tissue resident B cells, that may fine-tune the expression of Foxp3 in B-cell-induced regulatory T cells.

## Conclusions

To date, we know that naïve antigen-presenting B cell is sufficient to induce CD4^+^Foxp3^-^ regulatory T cells without additional cytokines or chemicals in an IL-10- and IL-27-dispensable and cell-cell contact-dependent manner. The expression levels of characteristic molecules differentiate Treg-of-B cells from well-known T helper and regulatory T cells as a brand-new type of CD4^+^Foxp3^-^ regulatory T cells (Fig. 1). Treg-of-B cells possess IL-10-depedent, IL-10-independent, and cell-cell contact-dependent suppressive abilities in antigen-specific and non-antigen specific fashions. Compared to long-term Treg-of-B cells, short-term Treg-of-B cells act through multiple suppressive pathways, and thus a blockade strategy would be more easily overcome through compensation by other pathways. Treg-of-B cells exhibit immunomodulatory effects in Th2-, Th1-, and Th17-medated diseases and even allogeneic transplantation. Nevertheless, the physiological conditions or cues necessary for Treg-of-B cell generation remain unknown. What is the fine-tuning mechanism for B cells to induce CD4^+^Foxp3^-^ or expand CD4^+^Foxp3^+^ T cells? What factors determine the kinetics, memory, and maintenance? And, most importantly, how could we use Treg-of-B cells in immunotherapy?

## Abbreviations

Breg: Regulatory B
Foxp3: Forkhead box P3
ICOS: Inducible T-cell co-stimulator
IL-10: Interleukin 10
iTreg: in vitro-induced Treg
LAG3: Lymphocyte-activation gene 3
PD1: Programmed cell death protein 1
pTreg: Peripherally derived regulatory T
TGF-β: Transforming growth factor-β
Th3: Type 3 helper
Tr1: Type 1 regulatory T
Treg: Regulatory T
Treg-of-B: B cell-induced regulatory T
tTreg: Thymus-derived regulatory T
